# Systemic amyloid A amyloidosis of the bladder after transurethral resection of urothelial carcinoma

**DOI:** 10.1002/iju5.12715

**Published:** 2024-03-04

**Authors:** Kaori Yamashita, Keita Yoshida, Tadao Nakazawa, Satoshi Kubota, Takahiro Shiseki, Eri Sekido, Masashi Inui

**Affiliations:** ^1^ Department of Urology Tokyo Women's Medical University Yachiyo Medical Center Yachiyo Japan; ^2^ Department of Pathology Tokyo Women's Medical University Yachiyo Medical Center Yachiyo Japan; ^3^ Department of Urology Graduate School of Medicine, Chiba University Chiba Japan

**Keywords:** amyloidosis, bladder, carcinoma

## Abstract

**Introduction:**

Amyloid A amyloidosis of the bladder is not a major disease. We report a patient with systemic amyloid A amyloidosis of the bladder after transurethral resection of urothelial carcinoma.

**Case presentation:**

An 87‐year‐old Japanese man had bladder carcinoma. He was followed up regularly with cystoscopy. Cystoscopy revealed multiple polypoid tumors 6 months after the first transurethral resection of urothelial carcinoma. Pathologic specimens contained the amyloid A component. He had hypertrophic cardiomyopathy, valvular disorders, and arrhythmias. His cardiac disease may have resulted from amyloid A amyloidosis. We speculated the patient had systemic amyloid A amyloidosis of the heart and bladder.

**Conclusion:**

We determined the type of amyloidosis via a biopsy of the bladder tumors. Our patient had cardiac disease. Therefore, systemic amyloid A amyloidosis could have caused his cardiac disease. The pathologic findings of bladder tumors can contribute to detecting systemic amyloid A amyloidosis.

**Abbreviations & Acronyms**
AA: amyloid A
AL: amyloid light‐chain
ATTRwt: wild‐type transthyretin amyloid
H&E: hematoxylin and eosin
KMnO_4_
: potassium permanganate
TUR‐Bt: transurethral resection of the bladder tumor

**Keynote message**

Systemic amyloid A amyloidosis of the bladder is uncommon. Our patient had amyloid A amyloidosis of the bladder after transurethral resection of urothelial carcinoma. He had hypertrophic cardiomyopathy, valvular disorders, and arrhythmias. His cardiac disease may have resulted from amyloid A amyloidosis. We diagnosed the patient with systemic amyloid A amyloidosis of the heart and bladder.

## Introduction

Amyloidosis is a rare and multisystemic disease. Amyloid deposits can be observed in multiple organs such as the heart, kidneys, liver, or gastrointestinal system.^1^ However, amyloidosis of the bladder is rare.

Major systemic types of amyloidosis include AL amyloidosis, ATTRwt amyloidosis, and AA amyloidosis. Ravichandran *et al*.^2^ reported that the frequency of AL amyloidosis, ATTRwt amyloidosis, and AA amyloidosis was 65.8% (7242/11 006 cases), 12.8% (1412/11 006 cases), and 8% (889/11 006 cases), respectively, in the United Kingdom from 1987 to 2019. AA amyloidosis is the third most common type in other organs; however, AA amyloidosis of the bladder is particularly rare.

## Case presentation

**Fig. 1:**
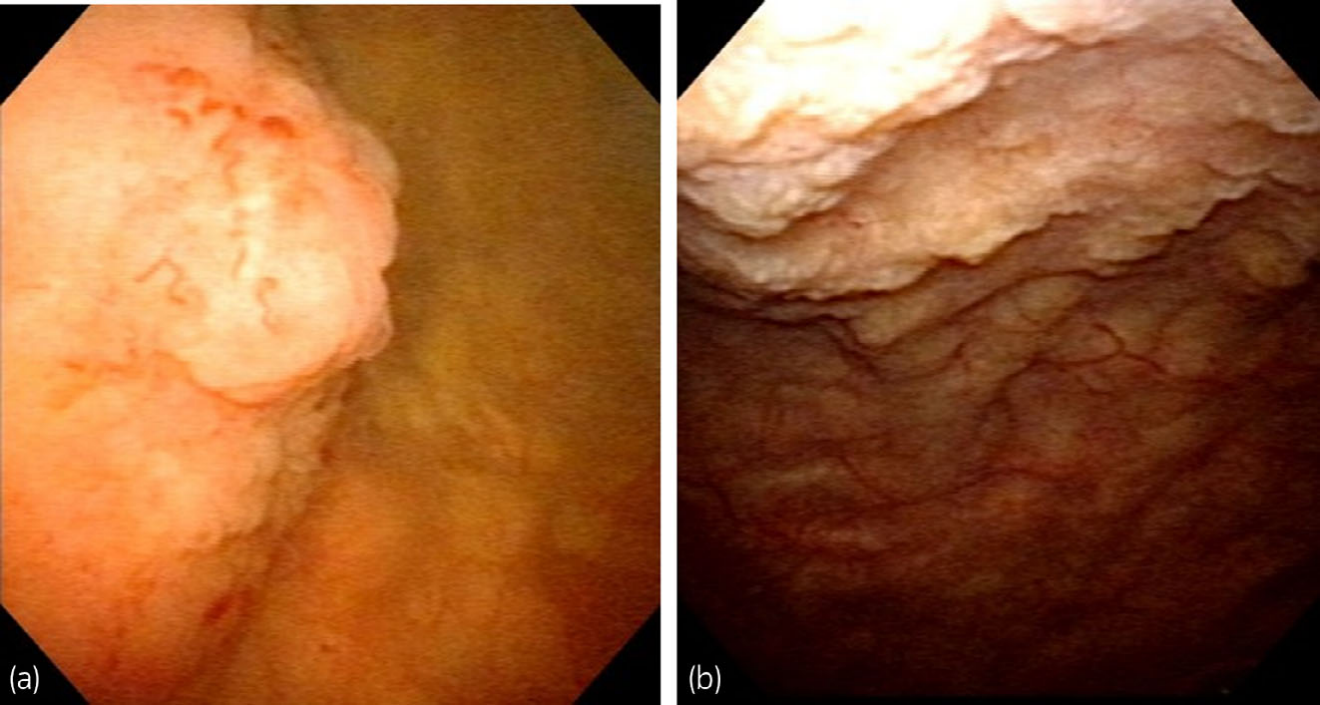
Imaging findings. (a) Cystoscopy shows a single sessile tumor, approximately 20 mm in diameter, that has developed on the right‐side wall of the bladder. (b) Cystoscopy shows multiple polypoid yellow tumors that developed on the anterior wall of the bladder.

An 87‐year‐old Japanese man presented with gross hematuria. Urine cytology was class IV. Cystoscopy revealed a single sessile tumor, approximately 20 mm in diameter, on the right‐side wall of the bladder (Fig. 1a). The bladder tumor was resected via transurethral resection. The pathologic result was high‐grade invasive urothelial carcinoma, stage pT1. The patient was followed regularly.

**Fig. 2:**
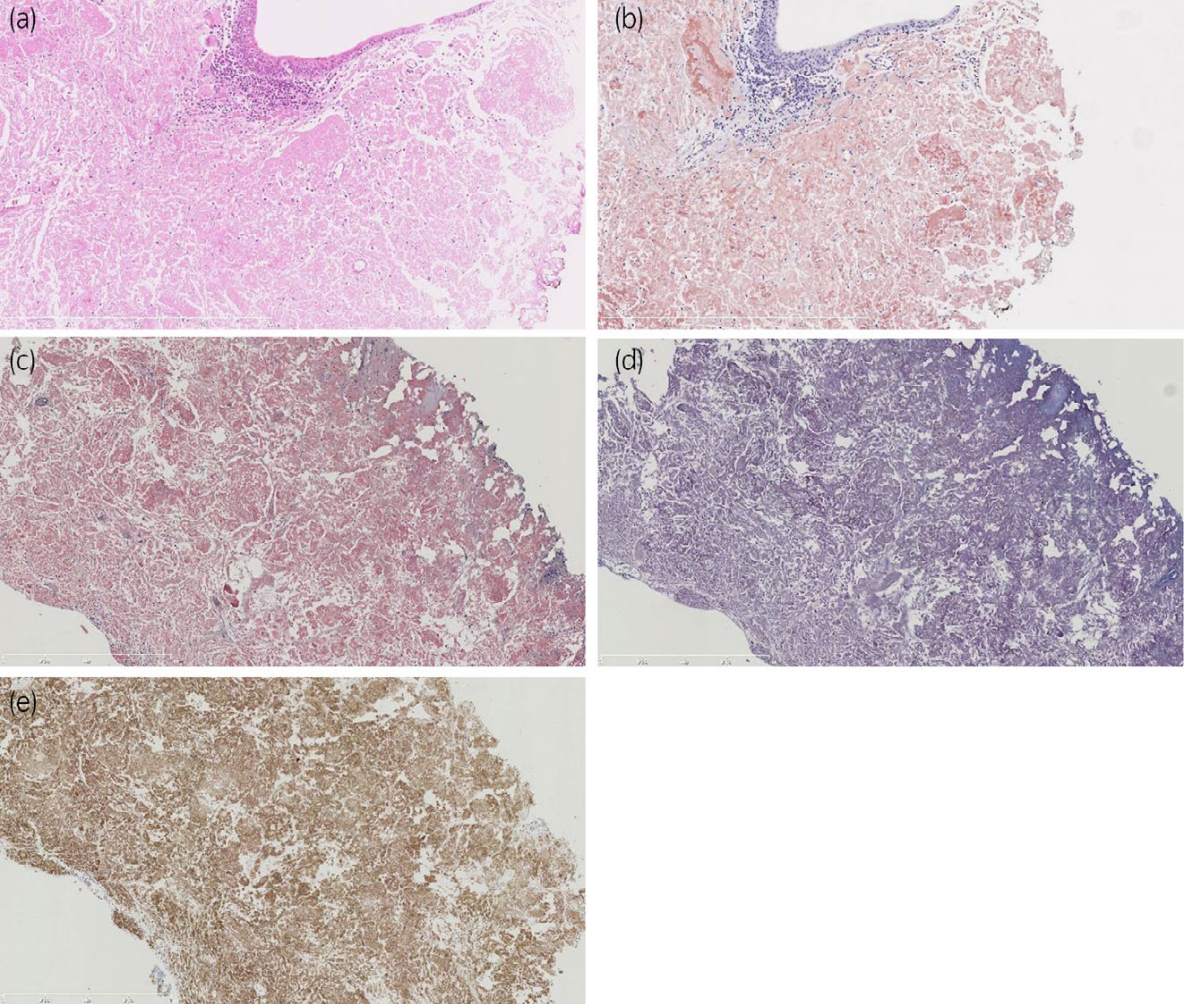
Microphotographs of the amyloid deposit. (a) An eosinophilic amorphous deposit is beneath the uroepithelium (H&E; magnification, ×40). (b) This area has few inflammatory or stromal cells (H&E; magnification, ×200). (c) The deposit is positive for Congo red staining (magnification, ×200). (d) The reactivity of the Congo red staining is lacking after subjecting the sample to the KMnO_4_ process (magnification, ×200). (e) The deposit immunohistochemically shows diffuse positivity for AA (magnification, ×200).

Cystoscopy revealed multiple polypoid yellow tumors that had developed on the anterior wall of the bladder 6 months after the first TUR‐Bt (Fig. 1b). This recurrence location was different from the previous surgery site. Urine cytology findings were negative. Computed tomography revealed no bladder tumor or any upper urinary tract abnormalities because the tumors were very small. TUR‐Bt was performed again. A deposit of eosinophilic amorphous material was beneath the nonneoplastic urothelium in the urinary bladder, based on H&E‐stained sections observed microscopically (Fig. 2a,b). In the Congo red‐stained section, the deposit's color ranged from red to orange under non‐polarized light, which suggested the presence of amyloid protein (Fig. 2c). Subjecting the section to the KMnO_4_ process resulted in the loss of reactivity for Congo red staining (Fig. 2d). Immunohistochemistry revealed that the deposit was positive for AA (Fig. 2e). We diagnosed the deposit as AA amyloidosis of the bladder. The evaluation of this patient's underlying diseases of AA amyloidosis revealed a C‐reactive protein level <0.03 mg/dL and no inflammatory conditions such as rheumatoid arthritis or uncharacterized inflammatory disease.

The heart is commonly involved in amyloidosis.^1^ The patient's electrocardiogram showed arrhythmias (e.g., nonsustained ventricular tachycardia and premature ventricular contractions), and echocardiography revealed fair left ventricular contraction and left ventricular hypertrophy, aortic valve stenosis and aortic regurgitation, and valve tricuspid regurgitation at the preoperative examination of the initial TUR‐Bt. After detecting AA amyloidosis of the bladder, we consulted his cardiovascular physician regarding the reason for his cardiac problem. We ultimately concluded that his cardiac condition was most likely caused by amyloidosis. The patient was diagnosed with systemic AA amyloidosis of the heart and bladder. However, a cardiac biopsy was not conducted owing to the patient's advanced age and cost. Therefore, we could not definitively determine a diagnosis of cardiac amyloidosis. At 12 months postoperatively, he had no recurrence of carcinoma or amyloidosis of the bladder.

## Discussion

Three major systemic types of amyloidosis exist: AL amyloidosis, ATTRwt amyloidosis, and AA amyloidosis.^1^, ^2^ AA amyloidosis can occur in the kidneys, liver, and heart.^1^ In a systematic review, Pyrgidis et al.^3^ reported 184 cases of amyloidosis of the bladder. They reported that the frequency of AL amyloidosis of the bladder and non‐AL amyloidosis of the bladder was 83.7% (154/184 cases) and 16.3% (30/184 cases), respectively. In the Pyrgidis study, 30 cases of non‐AL amyloidosis of bladder amyloidosis remained unclassified. Thus, the number of cases of AA amyloidosis of the bladder may be smaller.

AA amyloidosis is a secondary amyloidosis. Based on a nationwide survey conducted in Japan,^4^ the underlying diseases among patients with AA amyloidosis included rheumatic arthritis (60.3%: 120/199 cases), uncharacterized inflammatory disease (11.1%: 22/199 cases), and neoplasms (7.0%: 14/199 cases). To date, our patient has not had an underlying disease such as rheumatic arthritis. However, he had a history of urothelial carcinoma. The underlying disease with AA amyloidosis of our patient may be urothelial carcinoma.

Okuda *et al*.^4^ reported the clinical symptoms among their 199 patients with AA amyloidosis, which included cardiac failure (11.6% of patients) and atrial fibrillation (3.5% of patients). Our patient had cardiac disease and arrhythmias. His cardiovascular physician concluded that the patient had suspected comorbid cardiac amyloidosis and systemic AA amyloidosis. In patients with a clinical suspicion of cardiac amyloidosis, the electrocardiogram will show features of atrial fibrillation, ventricular arrhythmias, etc.^5^ The echocardiographic criteria for a diagnosis of cardiac amyloidosis include left ventricular thickness or valve thickness.^5^, ^6^ The clinical findings of our patient's electrocardiography and echocardiography were compatible with cardiac amyloidosis. A cardiac biopsy is an invasive test.^5^, ^6^ However, we believe that the pathologic analysis of a biopsy specimen of the bladder tumor was compatible with a definitive diagnosis of systemic amyloidosis.

Pyrgidis et al.^3^ recommend conducting cystoscopy at 3, 12, and 24 months after the initial TUR‐Bt. For a recurrence of localized amyloidosis of the bladder, treatment for the refractory disease is per os colchicine, intravesical 50% dimethyl sulfoxide, or partial or radical cystectomy. The treatment for systemic AA amyloidosis is the suppression of inflammation, and the choice of therapy depends on the nature of the underlying problem.^1^ For example, tuberculosis may be an underlying problem in AA amyloidosis and a patient would need antimicrobial therapy or rheumatologic disorders may be an underlying problem of AA amyloidosis and a patient would need biological therapy. However, chemotherapy or autologous stem cell transplant stands as the treatment for AL amyloidosis.^1^ Treatment is determined by the type of amyloidosis.^1^ Urologists should diagnose the type of amyloidosis via biopsy to choose a precise treatment.

## Conclusion

We were able to determine the type of amyloidosis via a biopsy of the bladder tumors. Our patient had cardiac disease and arrhythmias. Therefore, his cardiac disease was speculated to have resulted from systemic AA amyloidosis. The pathologic findings of bladder tumors could contribute to detecting systemic AA amyloidosis.

## Author contributions

Kaori Yamashita: Conceptualization; data curation; validation; writing – original draft; writing – review and editing. Keita Yoshida: Visualization. Tadao Nakazawa: Supervision. Takahiro Shiseki: Investigation. Eri Sekido: Data curation. Satoshi Kubota: Investigation. Masashi Inui: Supervision.

## Conflict of interest

The authors declare no conflicts of interest.

## Approval of the research protocol by an Institutional Reviewer Board

Not applicable.

## Informed consent

Written informed consent was obtained.

## Registry and the Registration No. of the study/trial

Not applicable.

## Funding information

There was no funding for this report.
